# The Role of m6A RNA Methylation-Related lncRNAs in the Prognosis and Tumor Immune Microenvironment of Papillary Thyroid Carcinoma

**DOI:** 10.3389/fcell.2021.719820

**Published:** 2022-01-03

**Authors:** Wenlong Wang, Cong Shen, Yunzhe Zhao, Botao Sun, Xiangyuan Qiu, Shujuan Yin, Jiaxin Chen, Xinying Li

**Affiliations:** ^1^ Thyroid Surgery Department, Xiangya Hospital, Central South University, Changsha, China; ^2^ National Clinical Research Center for Geriatric Disorders, Xiangya Hospital, Central South University, Changsha, China

**Keywords:** papillary thyroid carcinoma (PTC), tumor immune microenvironment, prognosis, m6A RNA methylation-related lncRNAs (m6A-lncRNAs), nomogram

## Abstract

Emerging evidence has indicated that N6-methylandenosine (m6A) RNA methylation plays a critical role in cancer development. However, the function of m6A RNA methylation-related long noncoding RNAs (m6A-lncRNAs) in papillary thyroid carcinoma (PTC) has never been reported. This study aimed to investigate the role of m6A-lncRNAs in the prognosis and tumor microenvironment (TME) of PTC. Three subgroups (clusters 1, 2, and 3) were identified by consensus clustering of 19 prognosis-related m6A-lncRNA regulators, of which cluster 1 is preferentially related to unfavorable prognosis, lower immune scores, and distinct immune infiltrate level. A risk-score model was established based on 8 prognosis-related m6A-lncRNAs. Patients with a high-risk score showed a worse prognosis, and the ROC indicated a reliable prediction performance for patients with PTC (AUC = 0.802). As expected, the immune scores, the infiltration levels of immune cells, and ESTIMATE scores in the low-risk subgroups were notably higher (*p* < 0.001) when compared with those in high-risk subgroups. Furthermore, GSEA analysis revealed that tumor associated pathways, hallmarks, and biological processes were remarkably enriched in the high-risk subgroup. Further analysis indicated that the risk score and age were independent prognostic factors for PTC. An integrated nomogram was constructed that accurately predicted the survival status (AUC = 0.963). Moreover, a lncRNA–miRNA–mRNA regulated network was established based on seven prognosis-related m6A-lncRNAs. In addition, 30 clinical samples and different PTC cells were validated. This is the first study to reveal that m6A-lncRNAs plays a vital role in the prognosis and TME of PTC. To a certain degree, m6A-lncRNAs can be considered as new, promising prognostic biomarkers and treatment targets.

## Introduction

In the past few decades, the incidence of thyroid cancer has sharply increased globally (Lim et al., 2017). Clinically, papillary thyroid carcinoma (PTC) is the most common histological subtype, accounting for up to 85% of all cases (Wang W. et al., 2020). Most patients with PTC usually present with indolent tumors and show a favorable prognosis after receiving standardized treatment. Nevertheless, up to 20–30% of patients with PTC experience recurrence or distant metastasis during follow-up (Wang W. et al., 2021; Sugino et al., 2020). Therefore, early detection and accurate management of the disease are vital for improving the prognosis. Unfortunately, the underlying molecular mechanisms that regulate PTC progression remain unknown.

N6-methylandenosine (m6A) modification is the most prevalent post-transcriptional epigenetic modification of mRNA or non-coding RNAs (ncRNAs) in eukaryotic cells that modulate RNA stability, translation, splicing, and export (Kasowitz et al., 2018; He et al., 2019; Ma et al., 2019). m6A modification is a reversible and dynamic process regulated by methyltransferases (m6A writers), binding proteins (m6A readers), and demethylases (m6A erasers) (Zhao Y. et al., 2020; Wang T. et al., 2020; Zhou et al., 2020). The methyltransferase complex includes *WTAP, METTL3, METTL14, RBM15, ZC3H13, KIAA1429*, and *RBM15B*, which mediate the methylation modification process. Demethylases consist of *ALKBH5* and *FTO*. The readers are composed of *IGF2BP1/2/3*, *YTHDF1/2/3, YTHDC1/2, HNRNPC,* and *HNRNPA2B1*. Increasing evidence has confirm that m6A modification is closely associated with embryonic stem cell self-renewal, immune response, tissue development, and ncRNA processing (Wen et al., 2018; Shulman and Stern-Ginossar, 2020; Yi et al., 2020). Recent studies have revealed that m6A modification also participates in the tumor occurrence and progression of various cancers, including hepatocellular, glioma, thyroid, colorectal, and breast cancers (Huang R. S. P. et al., 2020; Fang and Chen, 2020; Tian et al., 2020; Tu et al., 2020; Xu et al., 2020). For instance, the upregulated expression of *METTL3* in hepatocellular carcinoma is positively related to poor prognosis, while *METTL3*-mediated m6A modification led to epigenetic silencing of *SOCS2 via* an m6A-*YTHDF2*-dependent mechanism (Chen et al., 2018). Moreover, IGF2BP3 overexpression has been observed in colorectal cancer and the knockdown of IGF2BP3 has been shown to repress angiogenesis and DNA replication through reading m6A modification of *VEGF* and *CCND1*, respectively (Yang et al., 2020). In addition, the downregulated expression of *METTL14* in four breast cancer subtypes could predict unfavorable prognosis (Gong et al., 2020). These studies suggest that m6A regulators are highly involved in malignant biological processes, thus serving as useful therapeutic targets with promising prognostic values.

Several studies have highlighted that the aberrant expression of long non-coding RNAs (lncRNAs) also plays a critical role in cancer initiation and the development of PTC, and that the dysregulation of lncRNA is closely related to tumor development and progression (Fan et al., 2013; Xia et al., 2018). Another study has demonstrated that lncRNA GAS5 sponges miR-362-5p to upregulate *SMG1* toward promoting proliferation and invasion (Li L. et al., 2020). Upregulated lncRNA *MALAT1* levels exacerbate cell growth and invasion by regulating microRNA (miR)-204 (Ye et al., 2021). However, the underlying mechanism of m6A modification regulating the functions of lncRNA remains unclear. Therefore, so far, no study has elucidated the role of m6A methylation-related lncRNAs (m6A-lncRNAs) in the biological functions involved in PTC progression and tumor microenvironment (TME). Thus, a better understanding of the mechanisms of m6A-lncRNAs involved in PTC tumorigenesis and progression may help determine effective biomarkers that can precisely predict prognosis and develop personalized immunotherapy for PTC management.

In the present study, we systematically explored the prognostic significance and TME heterogenicity of m6A-lncRNAs in PTC. This study may provide new insight into the regulatory mechanisms involved in the tumor immune microenvironment and the treatment strategies for PTC.

## Materials and Methods

### Gene Datasets and Clinical Data Collection

**FIGURE 1:**
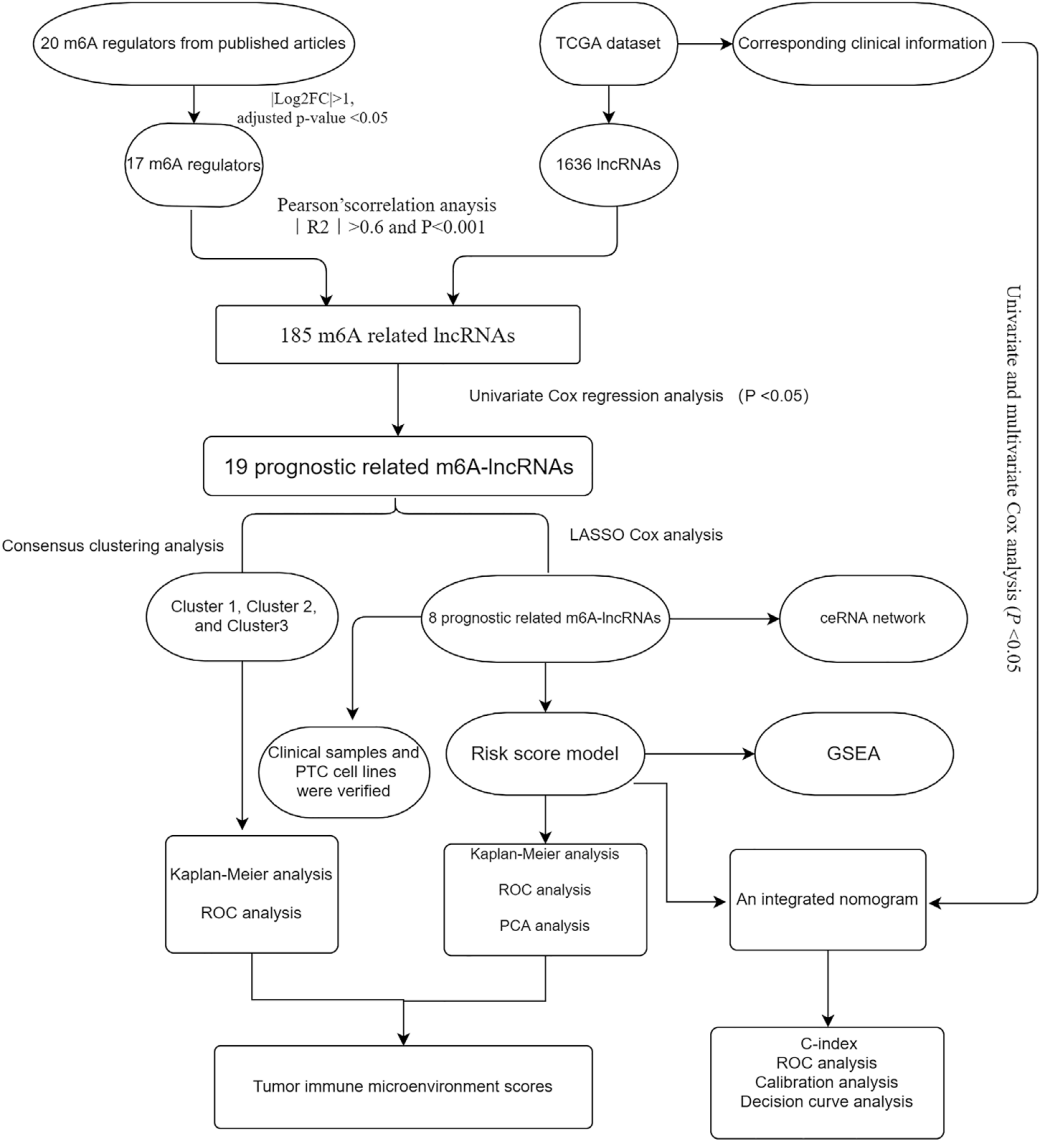
Study flow chart.

We downloaded the RNA-seq dataset containing 58 normal and 470 thyroid cancer samples from the TCGA database (https://portal.gdc.cancer.gov) with complete clinical information. The corresponding clinicopathological data including sex, age, multifocality, TNM stage, lymph node metastasis (LNM), histological type, extrathyroidal extension (ETE), bilaterality, and survival time were used for further analysis. According to previous publications, 20 m6A RNA methylation genes were identified, including writers (*WTAP, METTL3, METTL14, METTL16, KIAA1429, ZC3H13, and RBM15*), readers (*HNRNPA2B1, IGF2BP1/2/3, YTHDC1/2, FMR1, LRPPRC, and YTHDF1/2/3*), and erasers (*ALKBH5* and *FTO*). Next, the differential expression of these genes was assessed in the PTC versus normal samples by using the “Limma” package. The workflow of the present study is illustrated in Figure 1 **.**

### Risk Assessment Model Construction

First, Pearson’s correlation method was used to select the m6A related lncRNAs (m6A-lncRNAs) based on the threshold criteria of Pearson’s coefficient |R| >0.6 and *p* < 0.001, 185 lncRNAs were significantly associated with m6A RNA methylation genes. Then, univariate Cox regression analysis was performed to filter prognosis-related lncRNAs (*p* < 0.05). A total of 19 prognostic m6A-lncRNAs were extracted and analyzed. Subsequently, an unsupervised clustering algorithm by using the R package “Consensus-ClusterPlus (Wilkerson and Hayes, 2010)” was used to classify patients with PTC into different types of subgroups after conducting 1000 repetitions. Heatmaps were constructed by the Pearson distance measurement method and the average linkage method. To further identify the potential m6A-lncRNA regulators that affect prognosis, the least absolute shrinkage and selection operator (LASSO) Cox regression was performed to select the candidate risk m6A-lncRNA regulators. The risk score for each patient was calculated according to the following algorithm: Risk score $=\sum_{i=1}^{n}coefi*a\left(i\right)$ . The αi represented the expression level of m6A-lncRNAs, whereas coefi represents the coefficient of each m6A-lncRNAs.Thereafter, the patients were divided into low- and high-risk groups based on the median value of the risk score.

### Tumor Immune Estimation Resource

The immune and stroma scores for each patient were measured by the ESTIMATE algorithm (Yoshihara et al., 2013) *via* using the “estimate” R package with default parameters, and tumor purity was calculated based on genomic methods. The association between clustering subtypes and risk score, and the abundance of six types of infiltrating immune cells, including CD8^+^ T cells, macrophages, CD4^+^ T cells, dendritic cells (DC), B cells, and neutrophils, was calculated using the Tumor Immune Estimation Resource (TIMER) algorithm (Li T. et al., 2020).

### Enrichment Functional Analysis

Differentially expressed genes (DEGs) between the high- and low-risk subgroups were screened using the “limma” package based on *p* values < 0.05 and |log2FC| ≥1. Kyoto Encyclopedia of Genes and Genomes (KEGG) and gene ontology (GO) analysis (Kanehisa et al., 2016) was performed by using the “clusterProfiler” R package for pathway and functional enrichment analysis. On the other hand, gene set enrichment analysis (GSEA) (Mootha et al., 2003; Subramanian et al., 2005) was performed to identify the significant pathways in the high-risk subgroup when compared with that in the low-risk subgroup.

### CeRNA Network Construction

First, 39 miRNAs were extracted from the miRcode database based on 7 m6A-lncRNAs. Then, 72 mRNAs were identified by using miRTarBase (http://mirtarbase.mbc.nctu.edu.tw/php/index.php), miRDB (http://mirdb.org), and TargetScan (http://www.targetscan.org). Finally, a lncRNA–miRNA–mRNA competing endogenous RNA (ceRNA) network was created and visualized using the alluvial plot (http://www.bioinformatics.com.cn).

### Quantitative Real-Time PCR Validation

We collected 30 PTC tissue samples and paired the adjacent normal tissue samples from patients who underwent thyroidectomy in the Thyroid Surgery Department of Xiangya hospital from March 2020 to July 2020. The fresh tissues were stored at −80°C. Informed consent was obtained from all the participants and this study was approved by the Ethics Committee of Xiangya Hospital of Central South University. Furthermore, four human thyroid cancer cell lines (i.e., B-CPAP, K1, TPC-1, and IHH4) and human normal thyroid epithelial cell line (i.e., nthy-ori3-1) were cultured in a complete medium supplemented with 10% fetal bovine serum (Gibco, Carlsbad, United States) and RPMI1640 (Gibco) or DMEM (Gibco), supplemented with 100 U/mL penicillin (HyClone) and 100 mg/ml streptomycin. These cells were cultured at 37°C with 5% CO_2_ atmosphere.

Briefly, we used reverse transcription to construct the first strand of cDNA using 100 ng of total RNA according to the manufacturer’s instructions, Then, quantitative real-time PCR (qRT-PCR) analysis was performed using TBGreen Premix Ex TaqTMII (Cat # RR047A-5, TaKaRa, Japan). Primer sequences for m6A-lncRNAs are listed in Supplementary Table S1. Experiments were performed in triplicate.

### Statistical Analysis

Statistical tests were performed using SPSS 22.0 (IBM, NY, United States) and R version 3.6.0.

Chi-square and Student’s *t*-tests were performed to compare the differences between the two subgroups. The Kruskal–Wallis test was used to compare immune scores, stroma scores, tumor purity, and ESTIMATE scores among different cluster subgroups. Survival curves were depicted by using the Kaplan–Meier method. In addition, univariate and multivariate Cox regression analyses were performed to identify independent prognostic factors and establish an integrated nomogram combining predictable clinicopathological factors and risk scores. The predictive performance of the nomogram was validated by calibrated plots and the concordance index (C index). The receiver operating characteristic (ROC) curve was used to verify the prognostic ability of the nomogram for 3-/5-year OS, and a decision curve analysis was employed to assess the clinical values. The statistical significance was indicated as follows: *p* < 0.05 (*), *p* < 0.01 (**), *p* < 0.001 (***).

## Results

### The Profile of m6A -lncRNA Regulators

**FIGURE 2:**
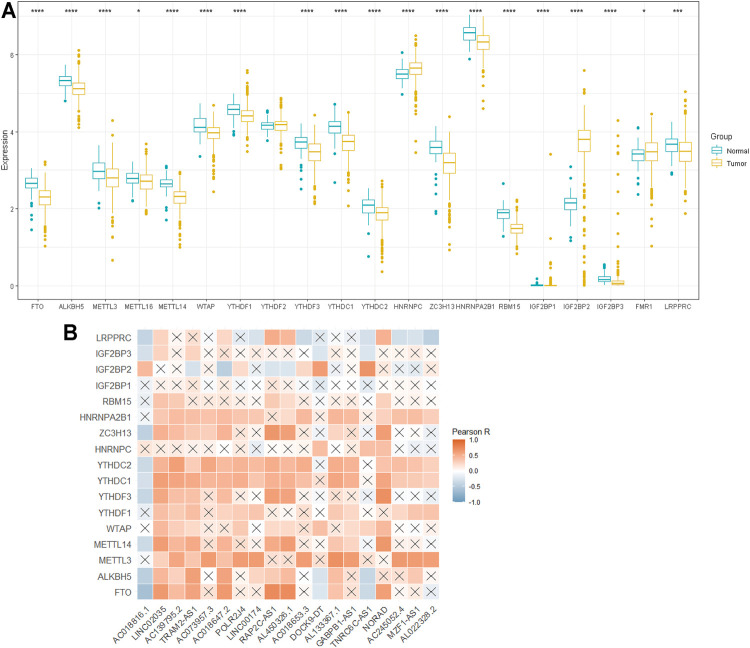
The profile of m6A -lncRNA regulators. **(A)**: Differential expression of 20 m6A RNA regulators; **(B)**: the correlations between m6A-related lncRNAs and m6A-related genes. *p < 0.05, **p < 0.01, and ***p < 0.001.

To determine the biological role of m6A-lncRNAs in the development of PTC, we first systematically explored the expression profiles of 20 m6A regulatory genes in PTC and the corresponding normal samples in the TCGA datasets. The expression of *ALKBH5, FTO, METTL3, METTL14, WTAP, YTHDF1, YTHDF3, YTHDC2, YTHDC1, ZC3H13, HNRNPA2B1, RBM15, IGF2BP1, IGF2BP3,* and *LRPPRC* was significantly downregulated in PTC than in the normal samples (*p* < 0.05), whereas that of *IGF2BP2* (*p* < 0.001) and *HNRNPC* (*p* < 0.001) was remarkably upregulated in PTC (Figure 2A). Figure 2B shows that most m6A regulators were positively correlated with the expression level of lncRNAs. These results indicated that m6A-lncRNA regulators are reliable factors for predicting prognosis.

### Consensus Clustering of m6A-lncRNA Regulators With Prognosis and the Tumor Immune Microenvironment

**FIGURE 3:**
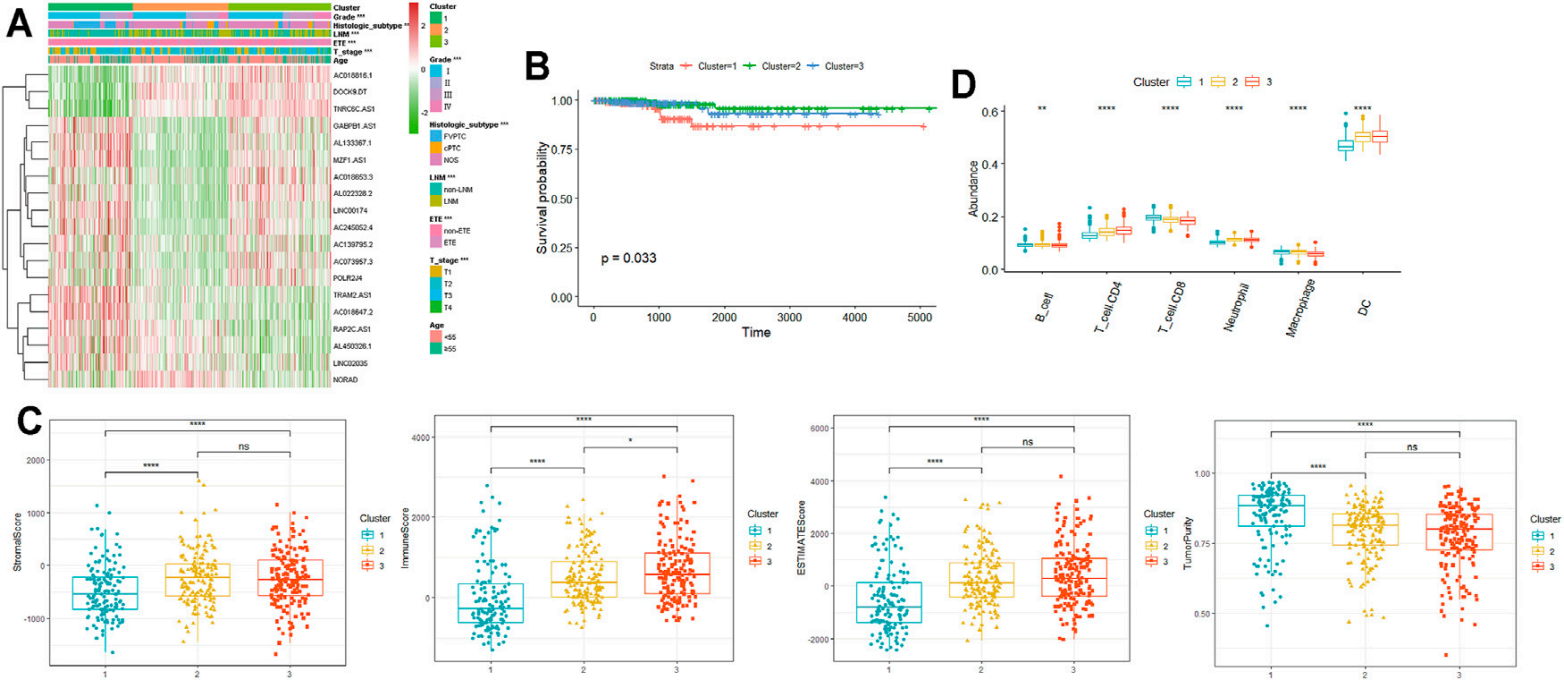
Prognosis and tumor immune microenvironment in consensus clustering. **(A)**: heatmap of cluster analysis clinicopathologic features; **(B)**: the OS of three subgroups; **(C, D)**: immunoscore and immune cell infiltration levels of cluster1/2/3 subtypes. OS: overall survival; LNM; lymph node metastasis; ETE: extrathyroidal extension. *p < 0.05, **p < 0.01, ***p < 0.001, and ns: no significance.

Next, according to the expression similarity of m6A-lncRNAs, k = 3 was considered as the optimal selection with the clustering stability increasing from k = 2 to 9 (Supplementary Figure S1). Therefore, a total of 470 PTC patients with complete clinical parameters were classified into three subgroups: cluster 1 (*n* = 141), cluster 2 (*n* = 158), and cluster 3 (*n* = 171). As shown in the heatmap of cluster analysis, 19 m6A-lncRNAs could be identified in different samples. We also found significant differences in the TNM stage, histological subtype, T stage, ETE, and LNM (all *p* < 0.001) among the three clusters (Figure 3A). Moreover, the OS of cluster 1 was significantly shorter than that of the other two clusters (*p* = 0.033, Figure 3B).

To better understand the effect of m6A-lncRNAs on the tumor immune microenvironment, we further evaluated the infiltration level of immune cells and immune scores among the three clusters. As shown in Figure 3C, ESTIMATE, immune score, and stroma score were markedly decreased, whereas the tumor purity score was significantly increased in cluster 1 than in the other two clusters (*p* < 0.001), which indicated that cluster 1 was characterized by reduced immune activity. Additionally, the abundance of B cells, CD8^+^ T cells, and macrophages was relatively higher, along with the relatively lower enrichment of CD4^+^ T cells, neutrophils, and DC in cluster 1 (Figure 3D).

### Risk Score Was Associated With Papillary Thyroid Carcinoma Prognosis and Tumor Immune Microenvironment

**FIGURE 4:**
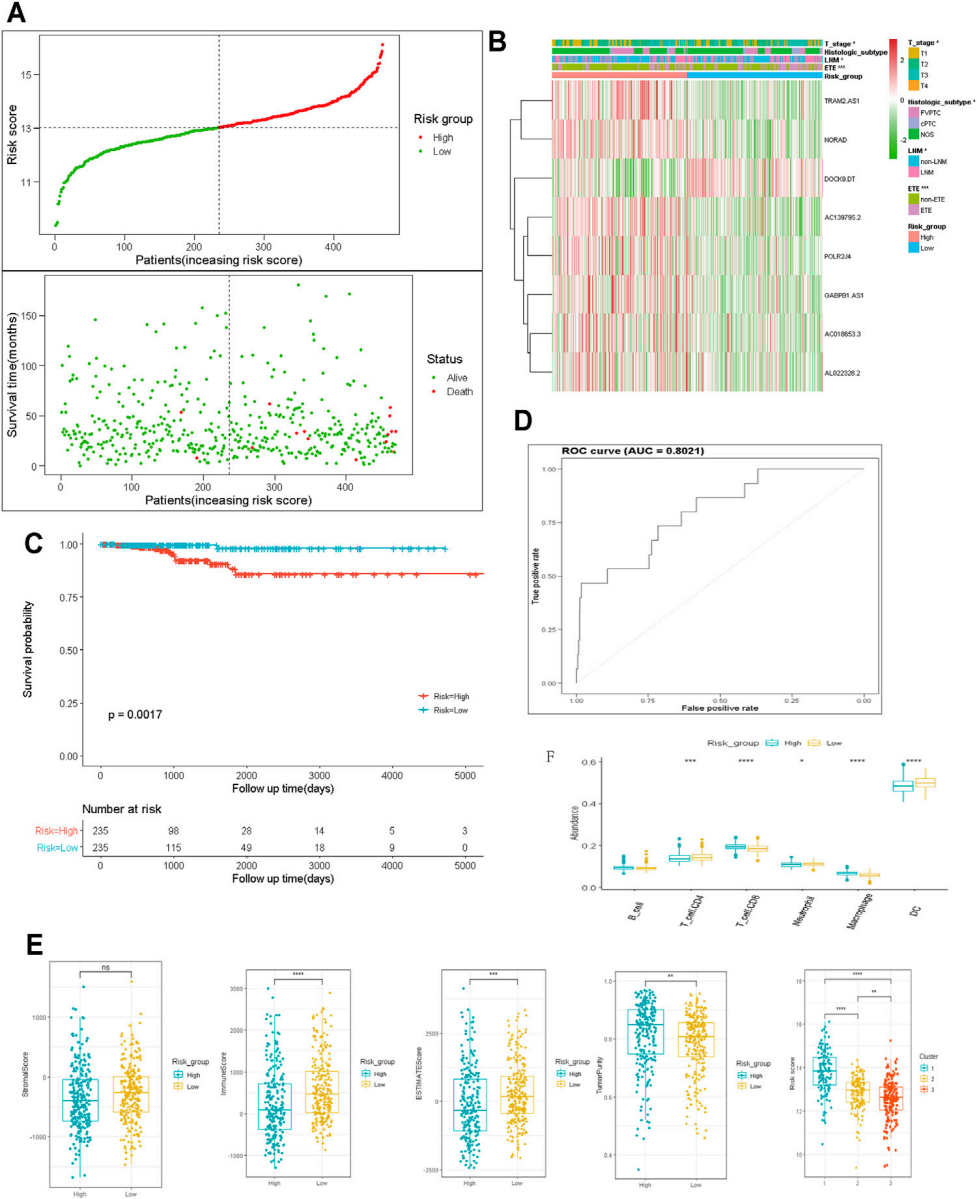
Construction and analysis of prognostic signatures of m6A -lncRNA regulators. **(A)**: the distributions of risk score, OS and OS status; **(B)**: the heatmap analysis the clinicopathologic features and eight m6A-lncRNA between low- and high-risk groups; **(C)**: Kaplan-Meier curves of OS by risk score group; **(D)**: the AUC value of the risk score; **(E, F)**: the immune cell infiltration landscape in the risk score subgroups. OS: overall survival; AUC: the area of ROC curve. LNM; lymph node metastasis; ETE: extrathyroidal extension. *p < 0.05, **p < 0.01, ***p < 0.001, and ns: no significance.

To establish the risk scores to predict the OS of patients with PTC, the LASSO Cox regression analysis was performed to further screen out prognosis-related m6A-lncRNAs, 8 m6A-lncRNAs exhibited strong prognostic value (Supplementary Figure S2). The risk score for each patient was calculated according to the following algorithm: Risk score = (0.536 × *AC139795.2*) + (0.131 × *TRAM2.AS1*) +(0.559 × *POLR2J4*) + (0.478 × *AC018653.3*) − (0.478 × *DOCK9.DT*) + (0.056 ×*GABPB1.AS1*) + (2.088 × NORAD) + (0.676 × AL022328.2). The patients were divided into low- and high-risk subgroups based on the median risk score. The principal component analysis could distinct the distribution of the two risk groups (Supplementary Figure S3), and the distributions of the risk score, OS, and OS status of each PTC patient are shown in Figure 4A. Heatmap distribution indicated that PTC patients with LNM, ETE, classical histological subtype, and T3-4 stage had higher risk scores (*p* < 0.05, Figure 4B). Survival analysis indicated that patients in the high-risk subgroup had worse OS compared with those in the low-risk subgroup (Figure 4C) and that the risk score model exhibited a good prediction performance with the area under the curve (AUC) of 0.8021 (Figure 4D), suggesting that the risk score model based on 8 m6A-lncRNAs could accurately predict the prognosis.

We also determined the relationship between the risk score and the tumor immune microenvironment. The ESTIMATE and immune scores were notably higher (*p* < 0.001), whereas the tumor purity score was significantly lower in the high-risk subgroup than in the low-risk subgroup. In cluster 1, which had a worse prognosis, the risk score was significantly higher than in the other two clusters (Figure 4E, *p* < 0.001). Moreover, the abundance of neutrophils, DCs, and CD4^+^ T cells were higher but that of CD8^+^ T cells and macrophages was distinctly lower in the low-risk subgroup than in the high-risk subgroup (Figure 4F). These data indicated that the tumor immune microenvironment plays a critical role in PTC tumorigenesis.

### GSEA, and Pathway and Functional Enrichment Analyses

**FIGURE 5:**
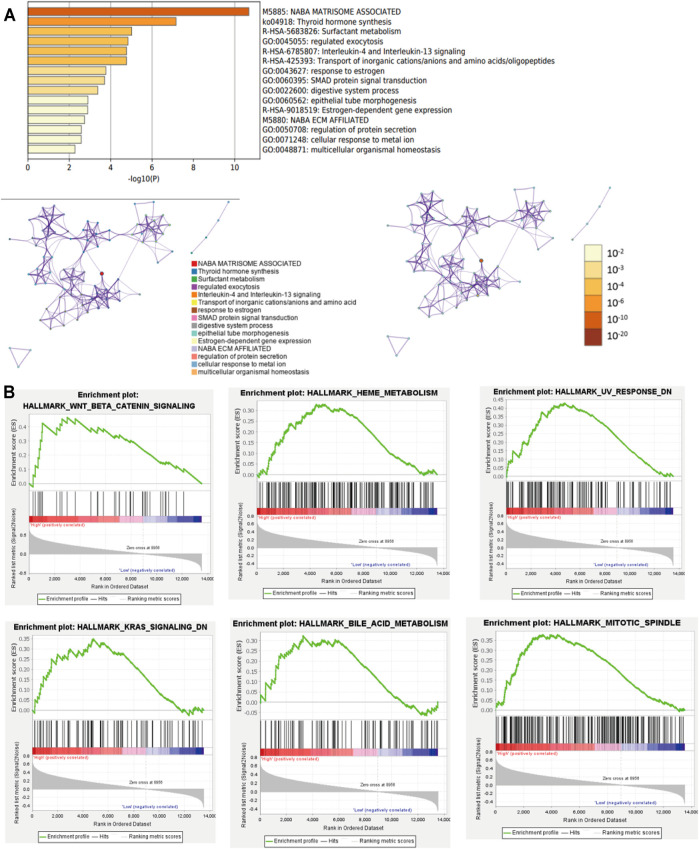
GSEA and Pathway and Functional enrichment analysis. **(A)**: KEGG pathway and GO function analysis; **(B)**: GSEA showed results in the high-risk group. KEGG: Kyoto Encyclopedia of Genes and Genomes; GO: Gene ontology; GSEA: Gene set enrichment analysis.

To better comprehend the potential biological mechanisms between the high- and low-risk score subgroups. The KEGG pathway and GO function analysis was implemented. The top 5 GO terms included NABA matrisome associated, thyroid hormone synthesis, surfactant metabolism, regulated exocytosis, and interleukin-4 and interleukin-13 signaling. (Figure 5A).

Furthermore, GSEA revealed that the malignant hallmarks of cancer, including Wnt/β-catenin signaling, HEME metabolism, UV response, KRAS signaling, bile acid metabolism, and MITOTIC spindle were closely associated with the high-risk subgroups (Figure 5B). These results cumulatively prove that the risk score was significantly associated with the biological mechanisms of PTC.

### Construction of a Prognostic Nomogram for Papillary Thyroid Carcinoma

We also implemented univariate and multivariate analyses to investigate the independent prognostic factors for PTCs. The forest plots revealed that the TNM stage (*p* < 0.01), age (*p* < 0.001), and risk sore (*p* < 0.001) were significantly correlated with OS in the univariate analysis, while the results of multivariate Cox regression analysis indicated that age (OR = 1.15; 95% CI, 1.08–1.22; and *p* < 0.001) and risk sore (OR = 2.16; 95% CI, 1.37–3.42; and *p* < 0.001) acted as independent prognostic factors (Table 1).

**TABLE 1:** Univariate and multivariate Cox regression analysis the prognosis factors of PTC.

Variable	Univariate analysis		Multivariate analysis	
	HR (95%CI)	*p*	OR (95%CI)	*p*
Age	1.17 (1.10–1.23)	<0.001	1.15 (1.08–1.22)	<0.001
Gender				
Male	ref		-	
Female	0.47 (0.17–1.32)	0.15	-	-
TNM stage				
Ι	ref		ref	
II	5.67 (0.80–40.20)	0.083	1.74 (0.23–12.99)	0.589
III	10.27 (2.13–49.50)	0.004	0.74 (0.13–4.18)	0.729
IV	14.19 (2.59–77.80)	0.002	1.67 (0.29–9.73)	0.567
Multifocality				
Unifocal	ref		-	-
Multifocal	0.28 (0.06–1.23)	0.092	-	-
Bilaterality				
Unilateral	ref		-	-
Bilateral	1.20 (0.26–5.44)	0.810	-	-
Isthmus	1.16 (0.15–8.95)	0.890	-	-
Risk score	4.23 (2.53–7.08)	<0.001	2.16 (1.37–3.42)	<0.001
Pathological type				
Other	ref		-	-
PTC	4.74 (0.62–36.10)	0.130	-	-

Abbreviations: CI: confidence intervals, PTC: papillary thyroid cancer.

**FIGURE 6:**
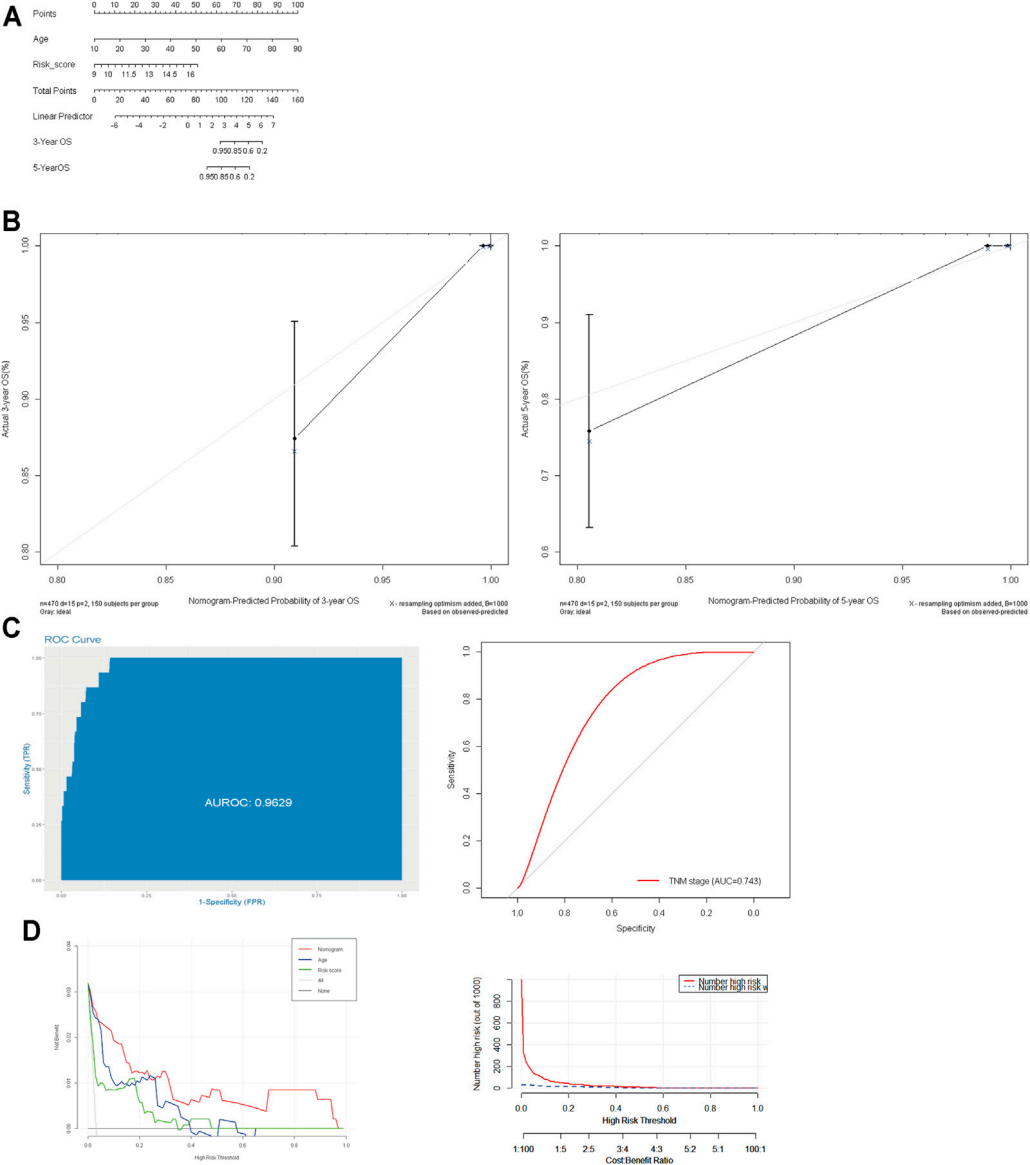
Construction and evaluation of prognostic nomogram model. **(A)**: Nomogram based on age and risk score for 3- and 5-year OS prediction; **(B)**: the calibration plots of nomogram model; **(C)**: the AUC value of the nomogram and AJCC TNM stage. **(D)**: decision curve analysis evaluated OS benefits. OS: overall survival.

Furthermore, to meet the requirement for clinicians to easily evaluate the prognosis of PTC patients, we formulated an integrated nomogram based on the independent prognostic factors for calculating the individual OS (Figure 6A). The C-index was 0.923, when compared with the TNM stage, and this nomogram model demonstrated better predictive performance (AUC: 0.743 vs.0.963) (Figure 6C). The calibration plots demonstrated good accuracy in predicting the 3- and 5-year OS (Figure 6B). Decision curve analysis (DCA) demonstrated that the integrated nomogram had an excellent net benefit when compared with the risk score model and age (Figure 6D). These data suggest that the nomogram can better predict the OS of patients with PTC.

### The ceRNA Network for Papillary Thyroid Carcinoma

**FIGURE 7:**
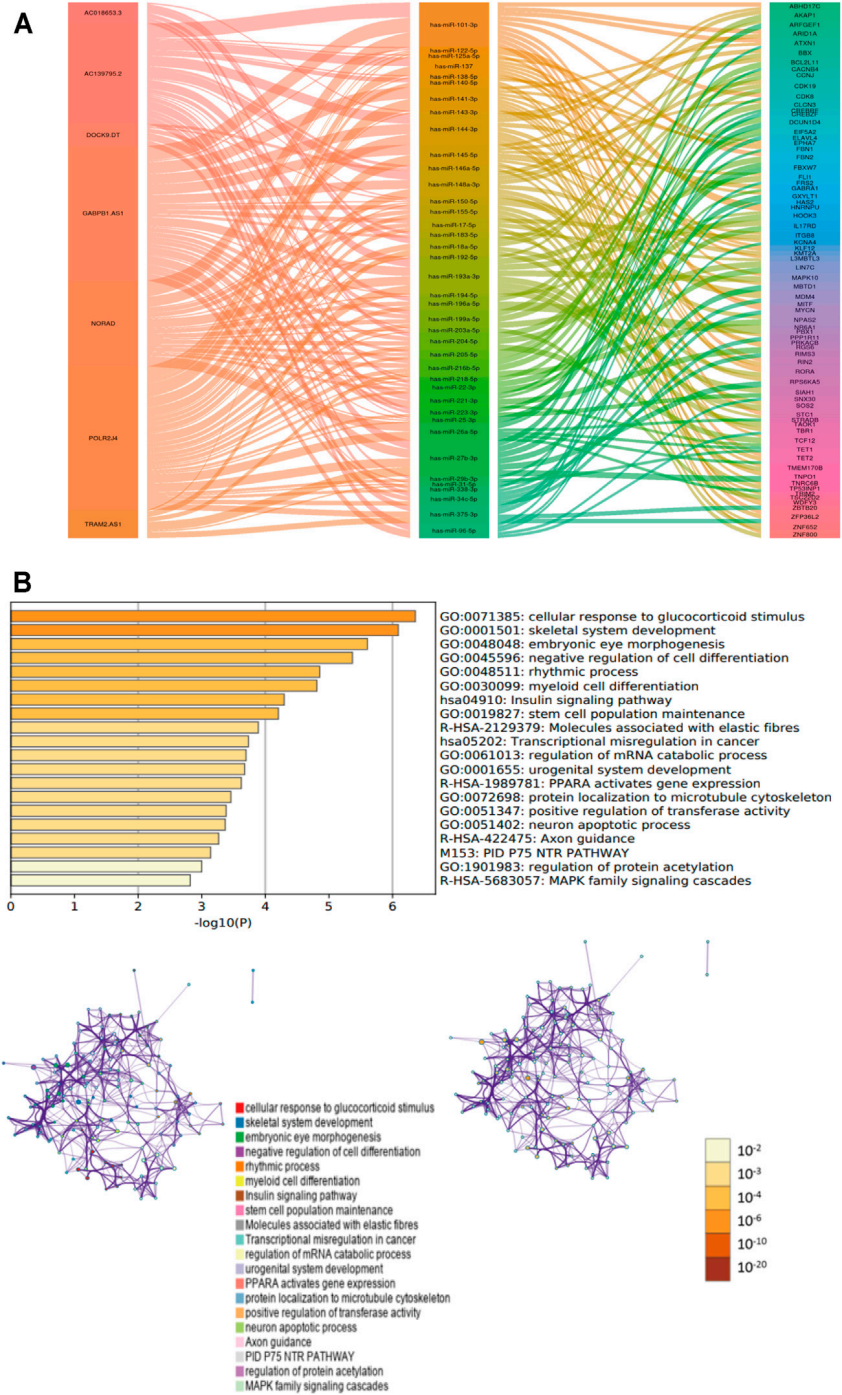
The ceRNA network for PTC. **(A)**: ceRNA network was constracted by seven m6A-lncRNA, thirty-nine miRNAs and seventy-two mRNAs. **(B)**: functional and pathways enrichment analysis for seventy-two mRNAs.

7 m6A-lncRNAs, 39 miRNAs, and 72 mRNAs were included in the ceRNA network (Figure 7A). Moreover, 72 target mRNAs were used to perform functional and pathway enrichment analyses, and the results indicated that these target genes were enriched in the cellular response to glucocorticoid stimulus, skeletal system development, embryonic eye morphogenesis, negative regulation of cell differentiation, rhythmic process, insulin signaling pathway, molecules associated with elastic fibers, and transcriptional misregulation in cancer (Figure 7B). These results may provide some potential insight into understanding the role of these m6A-lncRNAs in PTC tumorigenesis.

### Validation of m6A-lncRNAs Expression in Papillary Thyroid Carcinoma Tissue Samples

**FIGURE 8:**
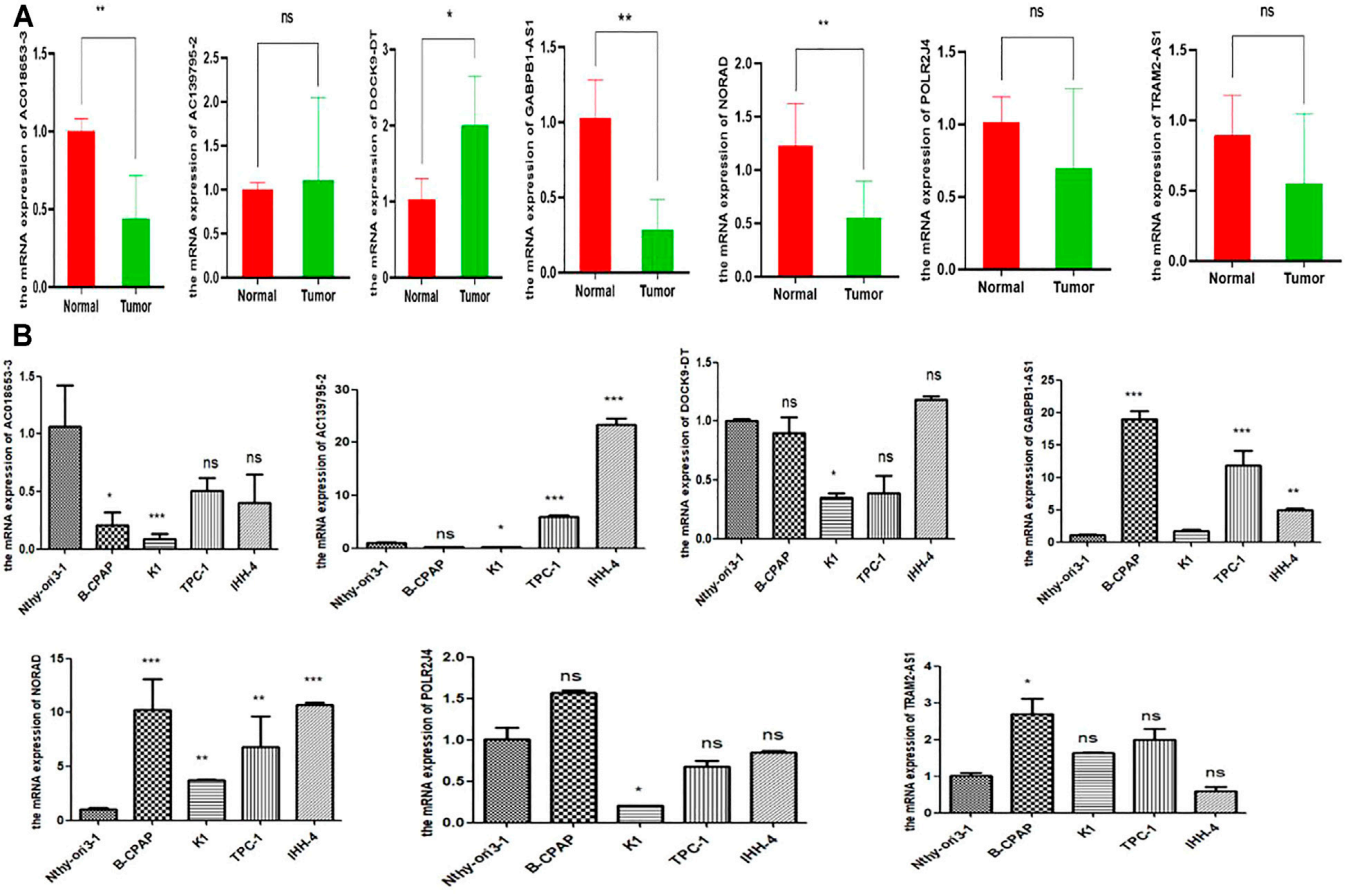
Validation the expression level of m5C-lncRNAs. **(A, B)**: the expression level of m5C-lncRNAs in 30 pairs PTC tissues and nine cells line. *p < 0.05, **p < 0.01, ***p < 0.001, and ns: no significance.

To validate the results of bioinformatics analysis, RT-qPCR was performed on PTC samples and cell lines. The expression level of *AC018653-3, GABPB1-AS1,* and *NORAD* were downregulated in PTC samples than in normal thyroid tissue samples, and only *DOCK9-DT* was significantly upregulated in PTC samples. *TRAM2-AS1, POLR2J4,* and *AC139795.2* revealed no significant differences (Figure 8A). Moreover, the expression level of *NORAD* and *GABPB1-AS1* were significantly upregulated in PTC cell lines than in normal thyroid epithelial cell (all *p* < 0.05). While *AC018653-3* and *AC139795.2* were significantly downregulated in PTC cell lines (both *p* < 0.05). However, the expression of *TRAM2-AS1, DOCK9-DT, and POLR2J4* showed no significant differences (Figure 8B).

## Discussion

The molecular pathogenesis and development of PTC have been attributed to various factors, including the abnormal expression of tumor suppressor genes and oncogenes, exposure to external radiation, and genetic mutations. m6A RNA methylation is a nascent field of research but is garnering considerable scientific attention. Increasing evidence suggests that m6A RNA methylation can target or modulate lncRNA to affect cancer initiation and development (Tu et al., 2020; Wen et al., 2020; Yi et al., 2020). To the best of our knowledge, this is the first study to systematically elucidate the potential contribution of m6A-lncRNA regulators in the prognosis of PTC and specifically highlight their role in the tumor immune microenvironment. Our findings provide a novel insight into the regulatory mechanisms that govern the tumor immune microenvironment based on which the treatment strategies for PTC can be developed.

Several studies have demonstrated that m6A modification plays a pivotal role in the pathological processes of carcinoma development (Wang J. et al., 2020; Guan et al., 2020; Zhang et al., 2020); however, its role in the lncRNA-dependent development of PTC remains unclear. In glioblastoma, *ALKBH5* interacts with the lncRNA *FOXM1-AS*, which enhances the demethylation of the 3′ UTRs of *FOXM1* transcripts to promote tumor proliferation and tumorigenesis (Zhang et al., 2017). In pancreatic cancer, *IGF2BP2* has been implicated as a reader to regulate lncRNA *DANCR*, leading to cell viability and proliferation, and stemness-like properties (Hu et al., 2020). *IGF2BP2* also directly binds to *PDX1* in an m6A-dependent manner and promotes pancreatic β-cell proliferation in type 2 diabetes (Regue et al., 2021). In this study, we performed Pearson’s correlation analysis to mine m6A-related lncRNA and identified three subgroups by consensus clustering: cluster 1, cluster 2, and cluster 3, respectively. These clusters not only affected the prognosis of PTC patients but were also closely associated with the TNM stage, histological subtype, T stage, ETE, and LNM (all *p* < 0.001). The incidence of LNM was significantly higher in cluster 1, as well as ETE, which indicated consensus clustering could effectively and accurately distinguish patients with poor prognosis. Moreover, patients with a high-risk score exhibited poor prognoses, PTC patients with LNM, ETE, and T3-4 stage simultaneously presented higher risk scores. These results correspond to the above analysis of consensus clustering, and cluster 1 was characterized by poor prognosis, which have higher risk score. This study fills the gap of m6A-lncRNAs signature in predicting the prognosis of patients with PTC and the risk stratification based on risk score could facilitate the determination of therapeutic options to improve prognoses.

Besides, the tumor immune microenvironment has received extensive attention so far. The TME is formed as a result of dynamic changes and is regulated by immune editing (Wang X. et al., 2021; Kaymak et al., 2021). The imbalance of TME can lead to the occurrence and development of diseases (Xie et al., 2020). The underlying mechanism of m6A modification on TME in PTC remains unclear. In this study, the risk score based on the eight m6A-lncRNAs were significantly correlated with immune cells infiltration. Compared with low-risk group, immune, stroma, and ESTIMATE scores were significantly downregulated in high-risk group. Moreover, the abundance of immune cells such as neutrophils, DCs, and CD4^+^ T cells was highly infiltrated in the low-risk group. As mentioned earlier, survival analysis confirmed that patients in cluster 1 had an unfavorable prognosis. Corresponding immune infiltration scores were decreased markedly, whereas the tumor purity score was significantly increased in cluster 1 compared to that in the other two clusters (*p* < 0.001). These findings indicated that the tumor immune microenvironment was significantly involved in the tumorigenesis of PTC.

Currently, the prognostic prediction and risk stratification for PTC patients is mainly dependent on the TNM scoring system, which is cumbersome and cannot accurately estimate the risk of recurrence (Haugen et al., 2016; Ghaznavi et al., 2018). Considering the heterogeneity in PTC, several strong prognostic biomarkers such as *BRAF* ^*V600E*^, TERT promoter, and RAS mutation have been widely reported (Zhao L. et al., 2020; Park et al., 2021). However, these potential biomarkers are not sufficiently sensitive and lack the accuracy in predicting the long-term survival rate in clinical practice. Although the incidence of PTC continues to rise, its mortality rate remains stable over 30 years, over diagnosis and over treatment of PTC have been new concerns (Pelizzo et al., 2004; Sugitani et al., 2021). Accurately assessing the prognosis for patients with PTC is critical to ensure that low-risk patients to avoid unnecessary I^131^ treatment and a higher degree of TSH inhibition, but that high-risk and advanced patients receive more aggressive treatments. Therefore, individualized treatment decision making can improve PTC prognosis and patient’s quality of life. To improve the accuracy of the survival prognostic model, we established an integrated nomogram by combining the predictable clinicopathological factors with the m6A-lncRNA risk scores. The calibration plots showed good accuracy in predicting the 3- and 5-year OS. Compared with TNM stage, our survival prediction model has better predictive performance (AUC: 0.743 vs. 0.963). In addition, to explore the role of m6A-lncRNAs in tumorigenesis and invasiveness of PTC, we constructed a ceRNA network based on the 7 m6A-lncRNAs, 39 miRNAs, and 72 mRNAs. An increasing number of studies reported that lncRNA, as ceRNA, involved in an indispensable role in different types of tumors, such as bladder cancer (Jiang et al., 2020), liver cancer (Wang et al., 2017), and breast cancer (Kong et al., 2019). Liu et al. (2018) demonstrated that LncRNA XIST negatively interacts with miR-34a to modulate the cell proliferation and invasion of PTC through MET-PI3K-AKT signaling. However, to date, research on m6A-lncRNA related ceRNA regulator networks in PTC is rare, which prompting might be a new research direction in future. Besides, GSEA revealed that the KRAS signaling and Wnt/β-catenin signaling were significantly enriched in the high-risk subgroups. He et al. (2021) disclosed that abnormal activation of the KRAS signaling could lower RNA methylation modification, which were related with poor prognosis in patients with breast cancer. Similarly, Han B. et al. (2020) demonstrated YTHDF1 as an amplifier of Wnt/β-catenin signaling to drive intestinal stemness. These studies have indicated that the mRNA of the KRAS signaling and Wnt/β-catenin signaling pathway molecule may serve as targets for m6A methylation modification.

Finally, we validated the mRNA expression of the prognostic m6A-lncRNAs in PTC samples and cell lines for subsequent functional and molecular experiments. Considering the expression level of m6A-lncRNAs, *NORAD*, and *GABPB1-AS1* were the most meaningful signatures for further research. Previous studies have demonstrated that *NORAD* promoted tumor proliferation and progression in non-small-cell lung cancer (Huang Q. et al., 2020), endometrial cancer (Han T. et al., 2020), and melanoma (Chen et al., 2019). In contrast, *NORAD* serves as a suppressor gene in neuroblastoma (Yu et al., 2020) and breast cancer (Liu et al., 2021), respectively, which is consistent with our results. Li and Wang (2021) reported *GABPB1-AS1* competitively bound to miR-330 and reinforced the *ZNF367* expression, thereby facilitating glioma cells progression. In cervical cancer, E6-induced GABPB1-AS1 overexpression facilitated tumor proliferation and invasion (Ou et al., 2020). However, the function and mechanism of *NORAD* and GABPB1-AS1 in thyroid cancer have not been reported, and its role in PTC needs further exploration.

Undeniably, there are several limitations in the present study. First, our findings are based on TCGA databases without our cohort, resulting in an inevitable selection bias in clinical and genetic data. Second, because of the limited project funding, we only used RT-qPCR to validate the level expression of m6A-lncRNAs, including cellular function- and regulation mechanism-based studies, are still needed. Third, the prognostic predictive model was based on the TCGA cohort with small sample size, and the interactions between the TME and m6A-lncRNAs are also not experimentally validated because of the lack of sufficient available datasets. Fourth, the correlation between m6A regulators and lncRNA has been analyzed, and there is a lack of experiments such as those using MeRIP-seq, m6A-IP-qPCR, and RNA-seq to further confirm m6A modification sites on lncRNA. Last, but not least, important clinical information, such as the treatment strategy (radioactive iodine ablation), TERT promoter and *BRAF* ^*V600E*^ mutation, and esophagus and tracheal invasions, was not available. Hence, future clinical and experimental studies are necessary to validate the application of our survival prediction model in clinical practice.

## Conclusion

In summary, this study systematically assessed the prognostic value, role in the TME, and potential regulatory mechanisms of m6A-lncRNAs in PTC. Three PTC subtypes were determined *via* consensus clustering and the risk score developed from 8 m6A-lncRNAs that stratified the prognosis and presented the significantly different TME. This is the first study to reveal that m6A-lncRNAs play a vital role in the prognosis and TME of PTC. To a certain degree, m6A-lncRNAs can be considered as new, promising prognostic biomarkers and treatment targets. Our findings also provide a crucial insight to support further research regarding the role of m6A-lncRNAs in PTC development.

## Data Availability

The original contributions presented in the study are included in the article/Supplementary Material, further inquiries can be directed to the corresponding author.
