# Supplementary material for: The Role of m6A RNA Methylation-Related lncRNAs in the Prognosis and Tumor Immune Microenvironment of Papillary Thyroid Carcinoma
Source: Front Cell Dev Biol. 2022 Jan 3;9:719820. doi: 10.3389/fcell.2021.719820 (PMC8762243; doi:10.3389/fcell.2021.719820)
Supplement: Supplementary file 1 [file Table1.DOCX]

Supplementary Material

Figure S1: The optimal cluster number was two using the ConsensusClusterPlus package.

Figure S2: LASSO analysis with minimal lambda value.

Figure S3: m6A-lncRNAs could be divided into two subgroups (green and red) based on the risk score.

Table S1: Quantitative real time PCR primers

| Primer Name | Primer sequence (5′-3′) | |
| --- | --- | --- |
| TRAM2-AS1 | Forward | GGCACAGTTCAGGTCCACAGTTA |
|  | Reverse | GTAAGGTGGAGTGCAGATTGAGG |
| POLR2J4 | Forward | AAGAGTCCGCTGTCTGTGATGTG |
|  | Reverse | TGTAGTCCTGAATGTCTGTTGGG |
| AC018653-3 | Forward | CCCGAATTTCACCTTAAACCAT |
|  | Reverse | ATTTCTGGCAGGCTTGGGA |
| DOCK9-DT | Forward | GAGCCTGGCAGATTGAAAGATAA |
|  | Reverse | CTCTGTCACCCAGCTCCACTCTT |
| GABPB1-AS1 | Forward | GTTGGGTAAAGAAGACAATGCTGG |
|  | Reverse | GTCCAAGTCATTACTGGCTCACA |
| NORAD | Forward | CACCCTCTGGGAAGATTTACTGG |
|  | Reverse | AGCTCCATGTCATCTAACCCACT |
| AC139795-2 | Forward | GCATTACAGTTTGGGAAGGTTCT |
|  | Reverse | ATGAGAATCACTTGAACCTGGGA |
