# Supplementary figures and images for: The Role of m6A RNA Methylation-Related lncRNAs in the Prognosis and Tumor Immune Microenvironment of Papillary Thyroid Carcinoma
Source: Front Cell Dev Biol. 2022 Jan 3;9:719820. doi: 10.3389/fcell.2021.719820 (PMC8762243; doi:10.3389/fcell.2021.719820)

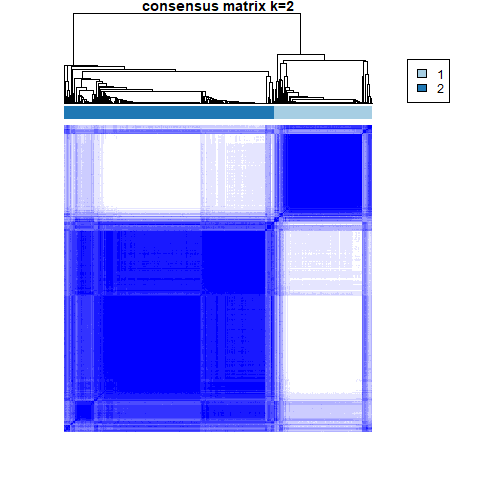

Supplement: Supplementary file 4 [file Image1.PNG]
